# Increasing Aspartoacylase in the Central Amygdala: The Common Mechanism of Gastroprotective Effects of Monoamine-Based Antidepressants Against Stress 

**DOI:** 10.3389/fphar.2022.823291

**Published:** 2022-02-25

**Authors:** Kaiyun Yao, Linyu Cao, Hongwan Ding, Yinge Gao, Tiegang Li, Guibin Wang, Jianjun Zhang

**Affiliations:** ^1^ Department of Pharmacology, Beijing, China; ^2^ State Key Laboratory of Bioactive Substance and Function of Natural Medicines, Institute of Materia Medica, Chinese Academy of Medical Sciences and Peking Union Medical College, Beijing, China

**Keywords:** monoamine-based antidepressants, water immersion restraint stress, gastric ulcer, N-acetylaspartic acid, central nucleus of amygdala, aspartoacylase

## Abstract

Monoamine-based antidepressants can prophylactically protect against stress-induced gastric ulcers. Although the central nucleus of amygdala (CeA) has been shown to modulate the severity of stress ulcers, little is known about the molecular mechanisms underlying the gastroprotective effect of this kind of drugs. Here, we first used proton magnetic resonance spectroscopy, a non-invasive tool, to explore the change of neurometabolites of the CeA of rats pretreated with the duloxetine of selective serotonin-norepinephrine reuptake inhibitors during 6 h of water-immersion restraint stress (WIRS). Duloxetine decreased **N-acetyl-aspartate/creatine ratio (NAA/creatine)** in CeA after WIRS, which was paralleled by the amelioration of gastric lesions. Meanwhile, the gastric ulcer index was negatively correlated with reduced NAA/creatine. Furthermore, the intra-CeA infusion of NAA aggravated WIRS-induced gastric mucosa damage, which suggested the crucial role of reduced NAA. Western blotting was performed to identify the specific enzymes responsible for the change of the contents of NAA at 0.5 h/3 h/6 h after WIRS, considering the preventative gastric protection of duloxetine. The NAA-catabolizing enzyme aspartoacylase (ASPA) was the only enzyme downregulated by 0.5 h WIRS and upregulated by duloxetine. Moreover, overexpressing ASPA in CeA alleviated stress ulcers. Additionally, all of the other three monoamine-based antidepressants, the fluoxetine of selective serotonin reuptake inhibitors, the amitriptyline of tricyclic agents, and the moclobemide of MAOs, increased ASPA expression in CeA. Together, these results indicate that increasing ASPA to hydrolyze NAA in CeA is a common mechanism of gastroprotective effects against stress exerted by monoamine-based antidepressants, and ASPA is a shared target more than monoamine regulation for this kind of drugs.

**GRAPHICAL ABSTRACT:**
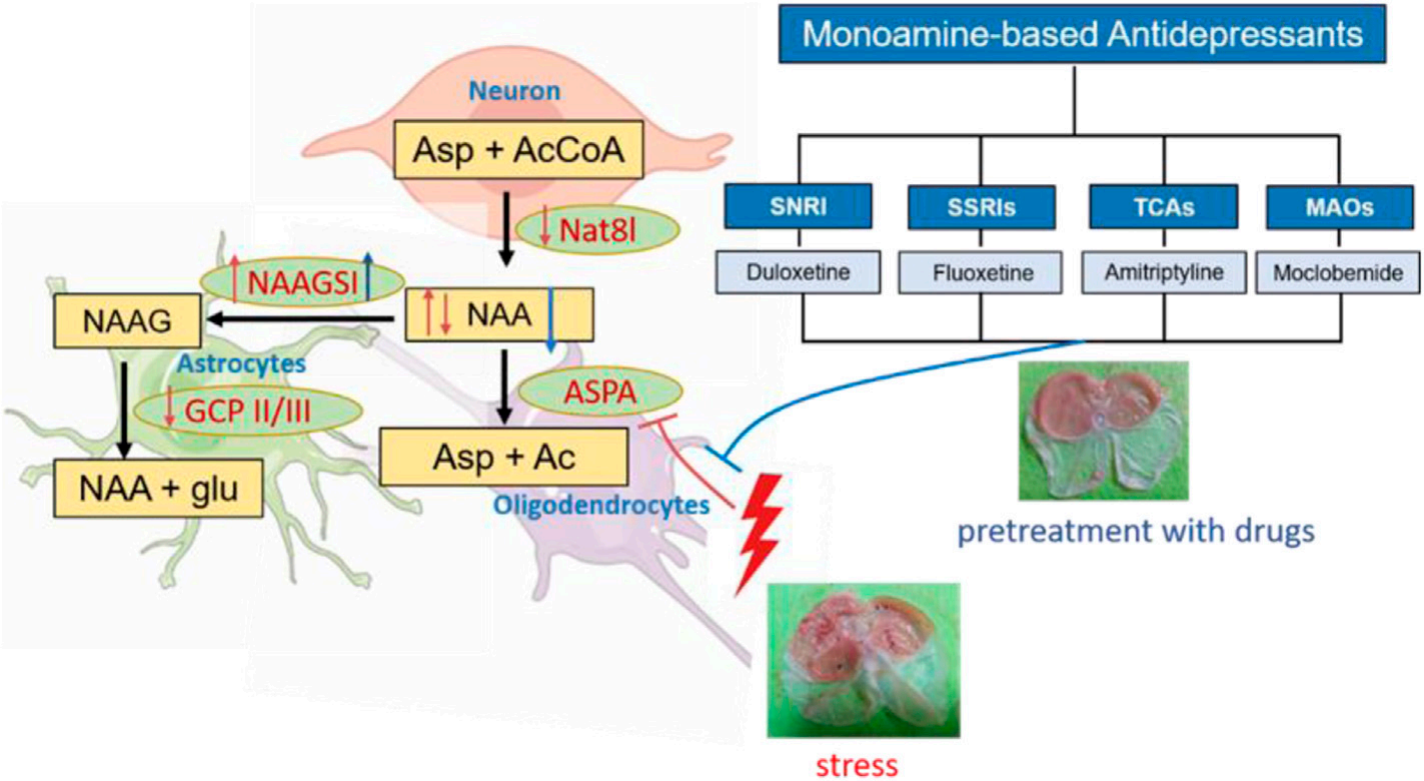


## Introduction

A disproportionate sensitivity to stress or excessive intensity of stressors may overwhelm the allostatic capacity, leading to poor adaptation, producing physiological and psychological abnormality, and resulting in organ damage and diseases such as gastric ulceration, depression, and posttraumatic stress disorder (McEwen 2008). Indeed, it is very important to carefully deal with mental disorders, in which several methodological approaches have been developed in this case (Bener et al., 2020; Güngör et al., 2020; Mirzaei and Nazemi 2022). Mucosal microbleedings and erosions or even gastric ulceration occurred as a typical stress-induced organ injury. Clinically, stress-ulcer bleeding is a severe complication with an estimated mortality of 40%–50% (Bardou et al., 2015). Pretreatment with multiple monoamine-based antidepressants such as the tricyclic agent (TCA) amitriptyline, selective serotonin reuptake inhibitors (SSRIs) fluvoxamine and fluoxetine, and the selective serotonin-norepinephrine reuptake inhibitor (SNRI) duloxetine has been shown to protect gastric mucosa damage caused by water immersion restraint stress (WIRS) (Khotib et al., 2019; Ji et al., 2012; Sen et al., 2002; Gabry et al., 2002). In spite of some findings, the understanding of the mechanisms of the gastroprotective effect against stress by antidepressants is mainly focused on the molecules of gastric tissue, and modulating events in the central nervous system (CNS) remain limited. The methods of drug discovery are crucial to find a new active compound for the medication of diseases and disorders (Zhao et al., 2021; Xu et al., 2021; Ghanadian et al., 2020).

Magnetic resonance spectroscopy (MRS) provides a non-invasive tool for investigating the longitudinal neurometabolic modulation in specific brain regions. This technology will help to explore the central mechanism of the prophylactic effect of the antidepressant on WIRS-induced gastric mucosa damage by comparing the possible differences of neurometabolites between the drug treatment and the vehicle group before and after stress, which was ignored in the most previous studies.

The enteric nervous system (ENS) and CNS are linked bidirectionally by the sympathetic and the parasympathetic pathways, forming the brain–gut axis (Goyal and Hirano 1996). Exposure to stress results in alterations of the brain–gut axis, which ultimately leads to the development of a broad array of gastrointestinal disorders including peptic ulcer (Konturek et al., 2011). The central circuitry activated by stress includes the paraventricular nucleus of the hypothalamus, amygdala, and periaqueductal gray, which sends an output to the automatic nerve system, hypothalamus–pituitary–adrenal axis (HPA), and pain modulatory system (Bhatia and Tandon 2005). Amygdala, especially central amygdala (CeA), has been shown to modify the severity of stress ulcer through an anatomical connection with hypothalamic and brainstem autonomic control areas, involved in the noradrenergic, dopaminergic, thyrotropin-releasing hormone and enkephalinergic mechanisms (Ray et al., 1990; Morrow et al., 1993; Glavin 1992; Ray and Henke 1991; Tanaka et al., 1998; Ray and Henke 1990). However, the molecular mechanisms in CeA involved in the gastroprotective effect of monoamine-based antidepressants against stress remain unclear.

Therefore, in the present study, we set out to explore the difference of metabolic alterations between SNRI duloxetine and vehicle using a longitudinal experimental design and examined whether the protective effect from the WIRS of duloxetine was associated with the difference of a certain neurometabolite by ^1^H-MRS. Based on the MRS results, we further identified the enzymes regulating the synthesis and degradation of the specific neurometabolite and examined the effects of the moclobemide of MAOs, amitriptyline of TCAs, and fluoxetine of SSRIs on the expression of enzymes to probe the potential common molecular mechanism of the gastroprotective effect against the stress of such kind of antidepressants. This study aimed to find a new target in the brain that can be intervened to prophylactically protect stomach mucosa from stress.

## Materials and Methods

### Drugs and Reagents

Duloxetine hydrochloride (Lilly Del Caribe, Inc., Puerto Rico), N-acetyl-L-aspartic acid (00920-5 G; Sigma, Shanghai, China), amitriptyline hydrochloride tablets (Changzhou Siyao Pharm, China), fluoxetine hydrochloride (Patheon, France), moclobemide (Adooq Bioscience, United States).

Primary antibodies were anti-Nat8l (Thermo, PA5-68424,1:250), anti-NAAGSI (Thermo, PA5-50959, 1:2,000), anti-GPCIII (BosterBio, A15227, 1:2,000), anti-aspartoacylase (ASPA) (Proteintech, 13244-1-AP, 1:2,000), and anti-GCPII (Proteintech, 13163-1-AP, 1:2,000). Anti-β-actin (Cell Signaling Technology, 8H10D10, 1:2,000) was used as a loading control. Secondary antibodies were goat anti-rabbit IgG (Proteintech, SA00001-2, 1:8,000) and goat anti-mouse IgG (Proteintech, SA00001-1, 1:8,000). Signal detection was performed using an ECL luminometer (GE Healthcare). Band optical densities were measured using ImageJ software. All experiments were performed at least twice.

### Animals and Housing

Male Sprague–Dawley rats (280–300 g) were obtained from SPF (Beijing) Biotechnology Co., Ltd. A total of 145 rats were used in the study. All animals were housed with a constant 12 h light cycle (lights on at 07:00) under controlled temperature (23 ± 1°C) and humidity (55 ± 5%). All animals had access to diet and water *ad libitum* before the experiments. All of the animal procedures performed in the present study were in accordance with the guidelines of the Animal Ethics Committee of Institute of Materia Medica, Chinese Academy of Medical Sciences.

### Water Immersion Restraint Stress

WIRS was carried out as previously described (Cao, et al., 2020). Briefly, rats were randomly divided into groups of 5–7. The rats received intraperitoneal injections of tested drugs (5 or 20 mg/kg duloxetine, 10 mg/kg fluoxetine, 10 mg/kg amitriptyline, 20 mg/kg moclobemide, and 50 mg/kg NAA) or vehicle 30 min prior to the stress. The animals were immobilized in perforated metal cages (length × width × height = 16 cm × 4 cm × 4 cm), which were vertically immersed in a bath with water at 16°C–18°C to the level of the animal xiphoid process. The procedure lasted up to 6 h. After modeling, all rats were executed and their stomachs were removed and injected with 10 ml of distilled water from the pylorus, followed by fixing in 4% formaldehyde for 15 min to count bleeding points as a measurement of the ulcer index; the ulcer length ≤0.5 mm is counted as 1, and the ulcer index is obtained by accumulating all ulcer counts in turn.

### Intra-CeA Infusion

Rats were anaesthetized using 5% chloral hydrate (350 mg/kg, i.p.) and placed in a stereotaxic apparatus. A double guide cannulae was placed above the CeA (AP: −2.9; ML: ±4.2; DV: −8.8; mm relative to bregma; AP, ML, and DV denote anteroposterior, mediolateral, and dorsoventral distance from the bregma, respectively). The coordinates were measured from the bregma according to the rat atlas. After surgery, rats were housed individually and fed *ad libitum* for 1 week.

NAA were freshly dissolved in ACSF (artificial cerebrospinal fluid) on the day of infusion. On the test day, obturators were removed and 5 μg/ul NAA at a volume of 1 μl or ACSF was delivered at a flow rate of 1 μl/min using 10 μl Hamilton syringes driven by a syringe pump. Injectors were left in place for additional 5 min to allow diffusion. Next, the obturators were placed back and the rats were put in the home house, and after 30 min, the WIRS procedure began.

### Adeno-Associated Virus

The recombinant adeno-associated virus (AAV) was designed and produced by Brain VTA, Wuhan, China. The AAV vector plasmid contains an expression cassette, which is composed of the EF1a promoter, cDNA encoding *ASPA* (GenBank accession number: NM_024399.1), and EGFP, connecting the element P2A peptide and WPRE and terminating element pA. The control virus is rAAV-EF1a-EGFP-WPRE-pA, which is also designed and produced by the company.

### Microinjection of Viruses

Rats were anesthetized with 5% chloral hydrate (**350** mg/kg, i.p.) for the bilateral stereotaxic injection of viruses into the CeA as before. We injected 600 nl of the virus into each location at a rate of 120 nl/min. The syringe was not removed until 15–20 min after the end of infusion to allow the diffusion of the virus and then slowly withdrawn. Following the delivery of the vectors, rats were quarantined for 72 h, followed by general housing for 3 weeks before WIRS.

### Verification of Viral Microinjection

Cells in CeA were infected with recombinant AAV containing sham or the ASPA expression cassette. Tissues were collected 3 weeks after infection, and protein was extracted for Western blot to confirm the infection efficiency by the expression of ASPA.

The location of virus injection was confirmed by a fluorescence microscope. Briefly, all rats receiving AAV-ASPA and the control virus were harvested for the brain to fix overnight in 4% PFA in PBS and transferred successively to phosphate-buffered salines (PBS, pH7.4) containing 20% and 30% sucrose before they were sliced with a microtome. The slices were mounted and then coverslipped on glass slides. The position of virus fluorescence expression was observed under a fluorescence microscope. Rats with “untargeted” injections were excluded from statistical analysis.

### Western Blot

Brain tissue was micropunched for CeA. The tissue was transferred to an RIPA protein extraction buffer (Beyotime Biotechnology, China) containing protease and phosphatase inhibitors (MedChemExpress, China), homogenized, and centrifuged at 12,000 g at 4°C for 15 min. The supernatant was separated, and protein concentration was measured using the BCA assay (Beyotime Biotechnology, China). Western blot was carried with the previously described methodology. Briefly, samples were separated using SDS-polyacrylamide gel electrophoresis and transferred to PVDF membranes (Millipore), which were then blocked with 5% non-fat milk in TBST for 1 h. The membranes were then incubated with primary antibodies against Nat8l/ASPA/GCP II/GCP III/NAAGSI and β-actin overnight at 4°C. After washing for three times in TBST, the membranes were incubated with horseradish peroxidase-conjugated anti-rabbit/mouse secondary antibodies in TBST for 1 h at room temperature and then visualized with an ECL luminometer. Quantitative analysis was performed with ImageJ software. All experiments were performed at least three times.

### ^1^H-MRS Acquisition and Spectral Analysis

A 7 T horizontal preclinical scanner (7 T Bruker PharmaScan 70/16 United States; Switzerland) was used to acquire ^1^H-MR single-voxel spectra. Animals were placed in a water-heated rat bed, and a nose cone was used to supply anesthesia. Rats were anesthetized during the experiments using isoflurane delivered in medical oxygen (O_2_). Physiological signs such as the respiration rate, body temperature, and ECG were continuously monitored over the time of the experiment using an animal monitoring system. The animals were placed in a prone position and then slid into the center of the magnet bore to obtain the imaging information of the rat brain axis (defined by the magnetic resonance scanning software, i.e., the section perpendicular to the head–tail line). The structural image scanning was carried as follows: T2-weighted imaging (T2WI) was performed to obtain the anatomical location information of the region of interest (ROI), which was CeA in our experiment. The scanning sequence is T2_TurboRare. Specific parameters are as follows: TR/TE = 5,300/33 ms, image size: 256 × 256, field of view: 35 × 35 mm, slice thickness: 0.56 mm, slices: 50, averages: 2, scan time: 5 min 39 s. Based on RARE scans, a localized spectroscopy voxel was located in the right CeA. The scanning sequence was PRESS_1H. Specific parameters: TR/TE = 2,500/18 ms. The voxel size was adjusted according to the size of the ROI. Spectrum data were analyzed by the instrument software Topspin 3.1.

### Statistical Analyses

All statistical analyses were performed with Graphpad Prism software version 7. Student’s t-test was used for comparing control and model groups, and one-way analysis of variance (ANOVA) followed by Tukey’s multiple comparison tests was used to test the model and drug-treatment groups. Correlation analysis was used to identify the association of the gastric ulcer index with NAA/creatine. Statistical significance (*p* < 0.05) was considered significant. The sample size for each experiment was reported in the figure legends. Each experiment was repeated at least three times.

## Results

Pretreatment with duloxetine reduced more NAA/creatine ratio in the right CeA, which was negatively correlated with lesions of gastric mucosa from WIRS.

**FIGURE 1:**
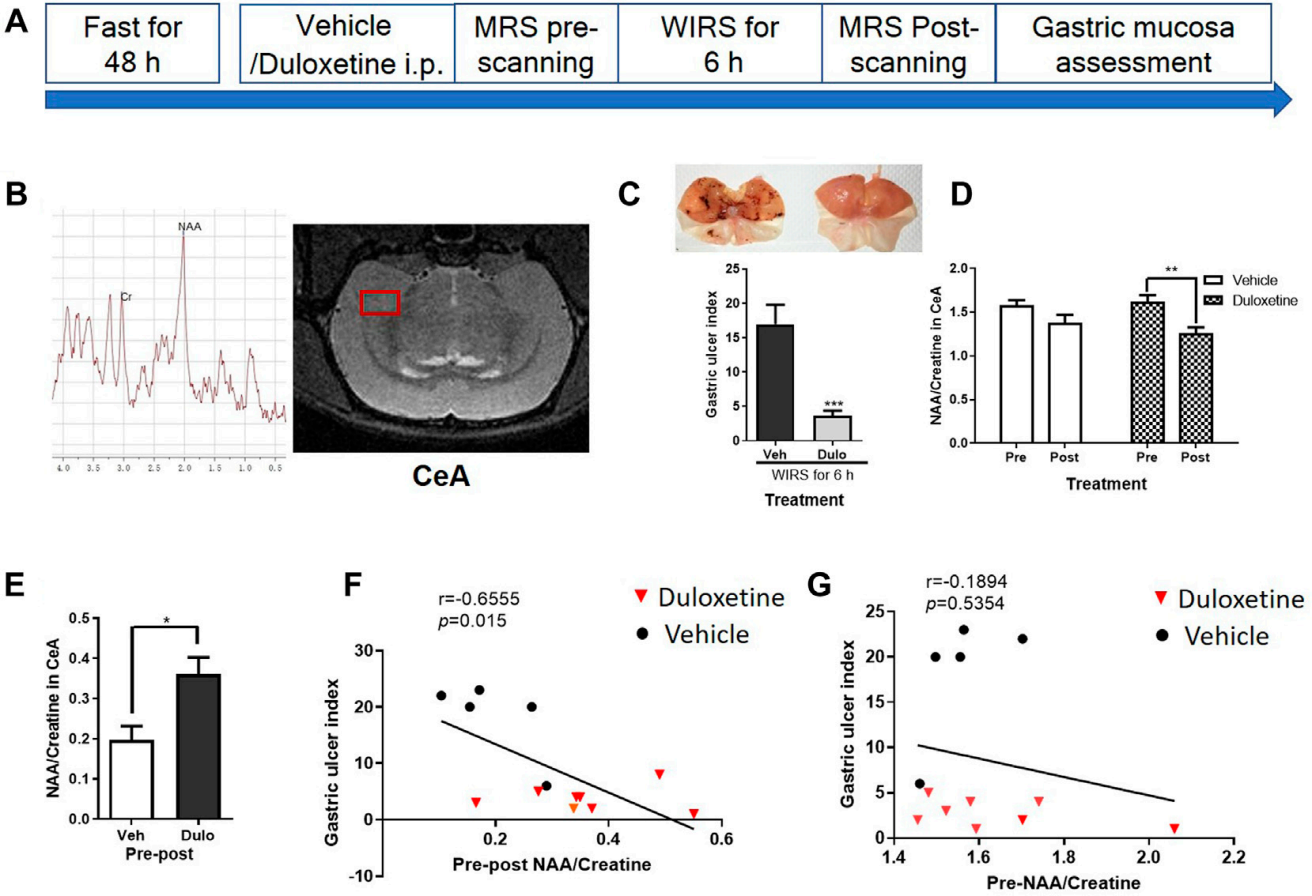
H-MRS showed difference of change of NAA/Cre ratio in the right CeA during WIRS between duloxetine and vehicle groups and correlation between gastric ulcer index and NAA/creatine in the right CeA when prophylactically protecting the gastric mucosa. **(A)** Illustration of the general timeline of the study. **(B)** Typical spectra images and schematic diagram of right CeA with voxel localization acquired for the rat. Right CeA was outlined by the red box. Each spectrum was evaluated for the peak area of NAA at 2.02 ppm, and creatine at 3.03 ppm. **(C)** Representative camera images of gastric mucosa and gastric ulcer index. (*n* = 5 in vehicle group, *n* = 8 in duloxetine group). ***p < 0.001, compared with vehicle group. **(D)** NAA/creatine ratio in the right CeA in pretreated vehicle and duloxetine groups before and after WIRS. *p < 0.05, compared with before WIRS of vehicle-treated group; ##*p* < 0.01, compared with before WIRS of duloxetine-treated group. (*n* = 5 in vehicle group, *n* = 8 in duloxetine group). **(E)** Change of NAA/creatine ratio in the right CeA between pre- and post-WIRS in the vehicle group was less than the duloxetine group. *p < 0.05, compared with change of NAA/creatine between post- and pre-WIRS in CeA of vehicle. **(F)** Relationship of change of NAA/creatine ratio in the right CeA with severity of gastric mucosa damage induced by WIRS for both vehicle and duloxetine groups. Significant linear correlation was observed between gastric ulcer index and change of NAA/creatine ratio in the right CeA for all the rats. **(G)** Relationship of NAA/creatine ratio in the right CeA 30 min after drug administration with severity of gastric ulcer damage induced by WIRS for both vehicle- and duloxetine-treated rats. No significant correlation was found between gastric ulcer index and NAA/creatine ratio in the right CeA for all the rats. All data are means ± SEM. Note: NAA, N-acetylaspartate; Veh: vehicle treatment group; Dulo: duloxetine treatment group; Pre: pre-WIRS; Post: post-WIRS.

Considering the length of MRI scanning, we just selected the right CeA as the interest of region (Figure 1B). The experiment was performed according to the timeline shown in Figure 1A. Pretreated duloxetine alleviated the gastric mucosa damage induced by 6 h of WIRS (Figure 1C, *p* < 0.001). There was a significant reduction of the N-acetyl-aspartate/creatine (NAA/Cre) ratio in the right CeA after WIRS in duloxetine groups (Figure 1D, *p* < 0.0001), while it was not the case in the vehicle group (*p =* 0.6063). There was no difference in the NAA/Cre ratio in the right CeA between two groups at 30 min after the drug administration, i.e., before WIRS (*p =* 0.4425). Furthermore, duloxetine-pretreated rats exhibited much more downregulation than vehicle-pretreated rats (Figure 1E, *p <* 0.05). Then, we performed linear regression to examine the relationship between the severity of gastric mucosa damage and the reduction of the NAA/creatine ratio in the right CeA. There was a significant negative correlation between the gastric ulcer index and change of NAA/creatine ratio in the right CeA for both the vehicle and duloxetine-treated rats (Figure 1F, *r* = −0.6555, *p* < 0.05). We also analyzed the relationship of the severity of gastric ulcer damage induced by WIRS with the NAA/creatine ratio in the right CeA 30 min after drug administration for both vehicle and duloxetine-treated rats. However, there was no significant correlation between the gastric ulcer index and the NAA/creatine ratio in the right CeA for all the rats at that time (Figure 1G, *r* = −0.1894, *p* = 0.5354).

**FIGURE 2:**
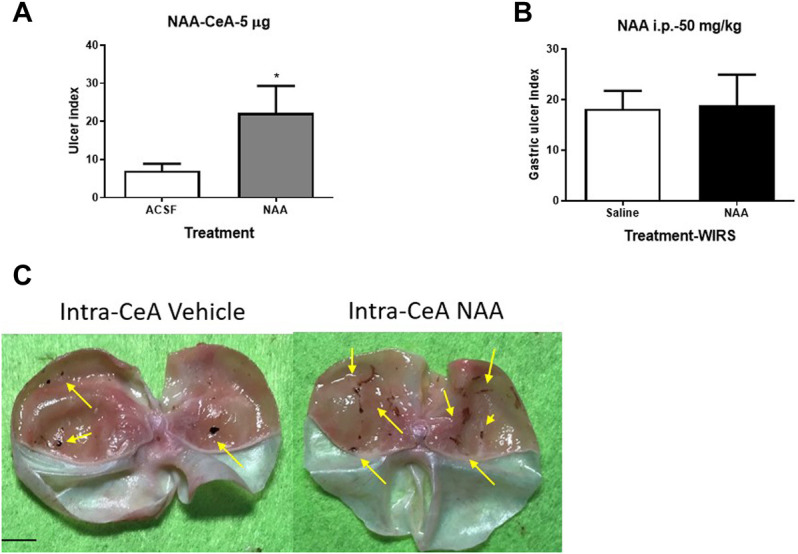
Effect of pretreatment with NAA on the WIRS-induced gastric ulcer. **(A)** Intra-CeA administration of 5 μg NAA aggravated WIRS-induced gastric mucosa damage. *p<0.05, compared with ACSF group. (*n* = 5 per group). **(B)** Intraperitonealadministration of 50 mg/kg did not affect WIRS-induced gastric ulcer. (*n* = 5 per group). **(C)** Representative camera images of gastric mucosa caused by water immersion with the treatment of intra-CeA of NAA or vehicle. The arrow indicates the ulcer position. Scar bar, 10 mm. All data are means ± SEM.

To clarify the relationship of the change of NAA content in the CeA and WIRS-induced gastric mucosa damage, we injected 5 μg NAA into the bilateral CeA and assessed the gastric ulcer index of rats after 6 h of WIRS. The NAA-treated group showed more serious gastric mucosa damage than the ACSF-treated group (Figures 2A,C, *p* < 0.05). NAA has been shown to induce oxidative stress and physiological abnormality in the stomach (Surendran 2009), so we tested whether the peripheral administration of NAA would produce a similar effect with intra-CeA administration. However, the i.p. administration of 50 mg/kg NAA did not affect the severity of gastric ulcer, following 6 h of WIRS (Figure 2B).

The effects of pretreatment with duloxetine on the expression of enzymes involved in synthesizing and metabolizing NAA: ASPA/GCP II/GCP III/NAAGSI/Nat8l in the CeA after 6 h/3 h/0.5 h of WIRS.

**FIGURE 3:**
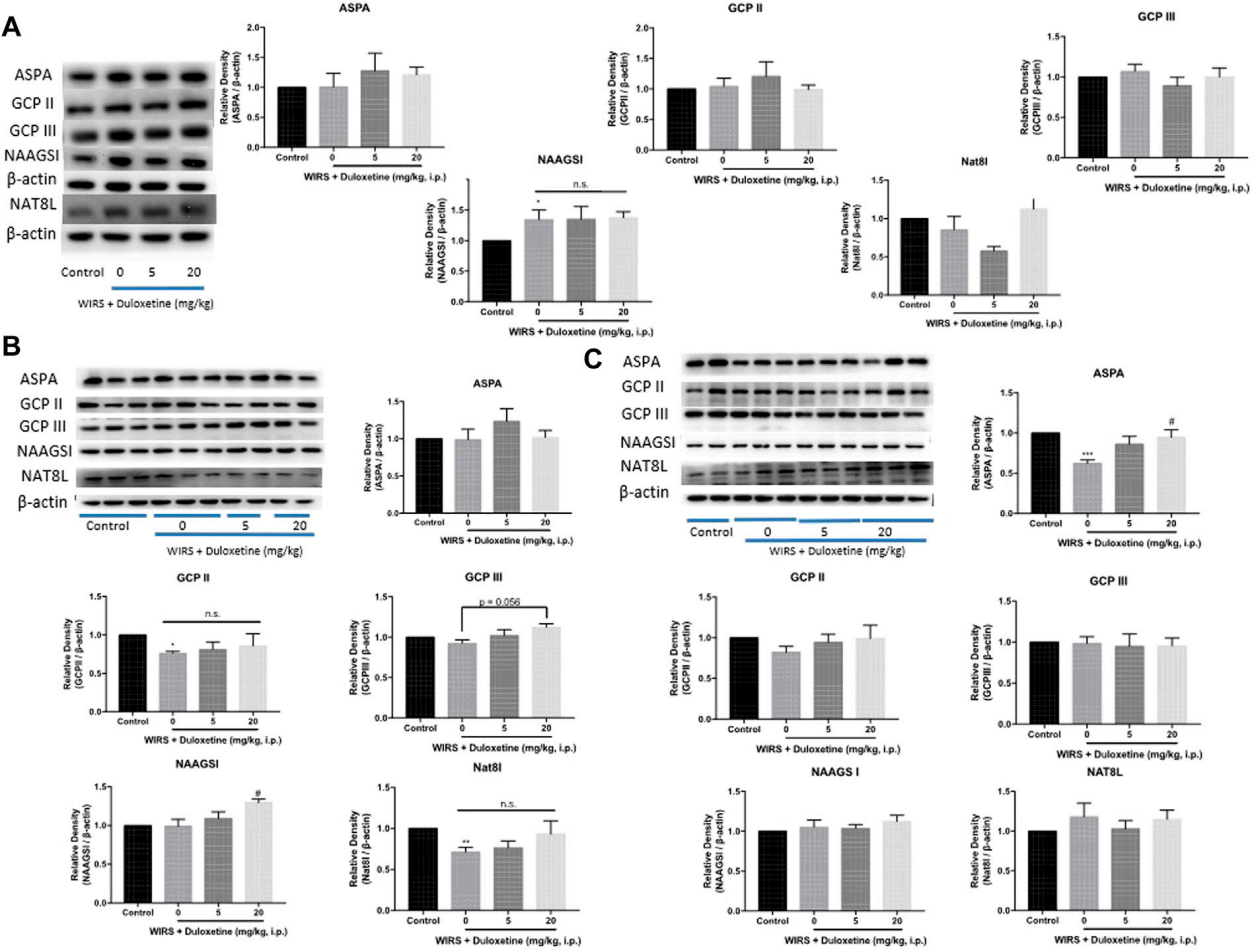
Effect of pretreatment with duloxetine on NAA-related enzyme expression in CeA in rats exposed to 6 h/3 h/0.5 h of WIRS. **(A)** Representative immunoblots of ASPA/GCP II/GCP III/NAAGSI/Nat8l performed in CeA lysates. Western blotting showed that duloxetine pretreatment had no effect on the expression level of these enzymes in CeA after 6 h of WIRS (*n* = 5 in control, 5 mg/kg duloxetine and 20 mg/kg duloxetine group, *n* = 6 in 0 mg/kg duloxetine group). **(B)** WIRS for 3 h decreased the expression of GCP II and Nat8l in CeA, and duloxetine did not modulate the expression of these two enzymes (*n* = 5 per group), while pretreatment with 20 mg/kg duloxetine increased NAAGSI and slightly increased GCP III expression in CeA exposed to WIRS, which did not change by WIRS (*n* = 6 per group). **(C)** WIRS for 0.5 h decreased the expression of APSA in CeA, and pretreatment with 20 mg/kg duloxetine reversed this decrement (*n* = 6 per group). ASPA/GCP II/GCP III/NAAGSI/Nat8l was normalized to β-actin. Values are expressed as means ± SEM. *p < 0.05, **p < 0.01,***p < 0.001, compared with control group, compared with control group, #*p* < 0.05, compared with WIRS + vehicle group.

Firstly, we examined the expression of ASPA/GCP II/GCP III/NAAGSI/Nat8l in the CeA after 6 h of WIRS to determine which enzyme was responsible for the reduction of NAA. Western blotting showed that the NAAGSI expression was higher in all the groups exposed to WIRS for 6 h, whether they were pretreated with duloxetine or not. However, the other four enzymes did not change after 6 h of WIRS, and both 5 and 20 mg/kg duloxetine did not affect the expression (Figure 3C). Since duloxetine was administered prophylactically, we inferred that the expression of the enzyme regulating NAA reduction initiated by duloxetine may change already and return to the normal level at 6 h. We forwarded the testing time to 3 and 0.5 h after the beginning of WIRS. Western blotting showed that WIRS for 3 h decreased the expression of GCP II and Nat8l in CeA (Figure 3B, *p* < 0.05), while duloxetine did not affect these two enzymes. Although NAAGSⅠand GCP Ⅲ in CeA were not regulated by 3 h of WIRS, 20 mg/kg duloxetine increased the expression of NAAGSⅠ (*p* < 0.05) and marginally upregulated GCPⅢ (*p* = 0.056).

However, only ASPA was decreased after 0.5 h of WIRS (Figure 3C, *p* < 0.05), and 20 mg/kg duloxetine increased the expression of ASPA in CeA significantly (*p* < 0.01). The other four enzymes did not differentiate among the four groups.

**FIGURE 4:**
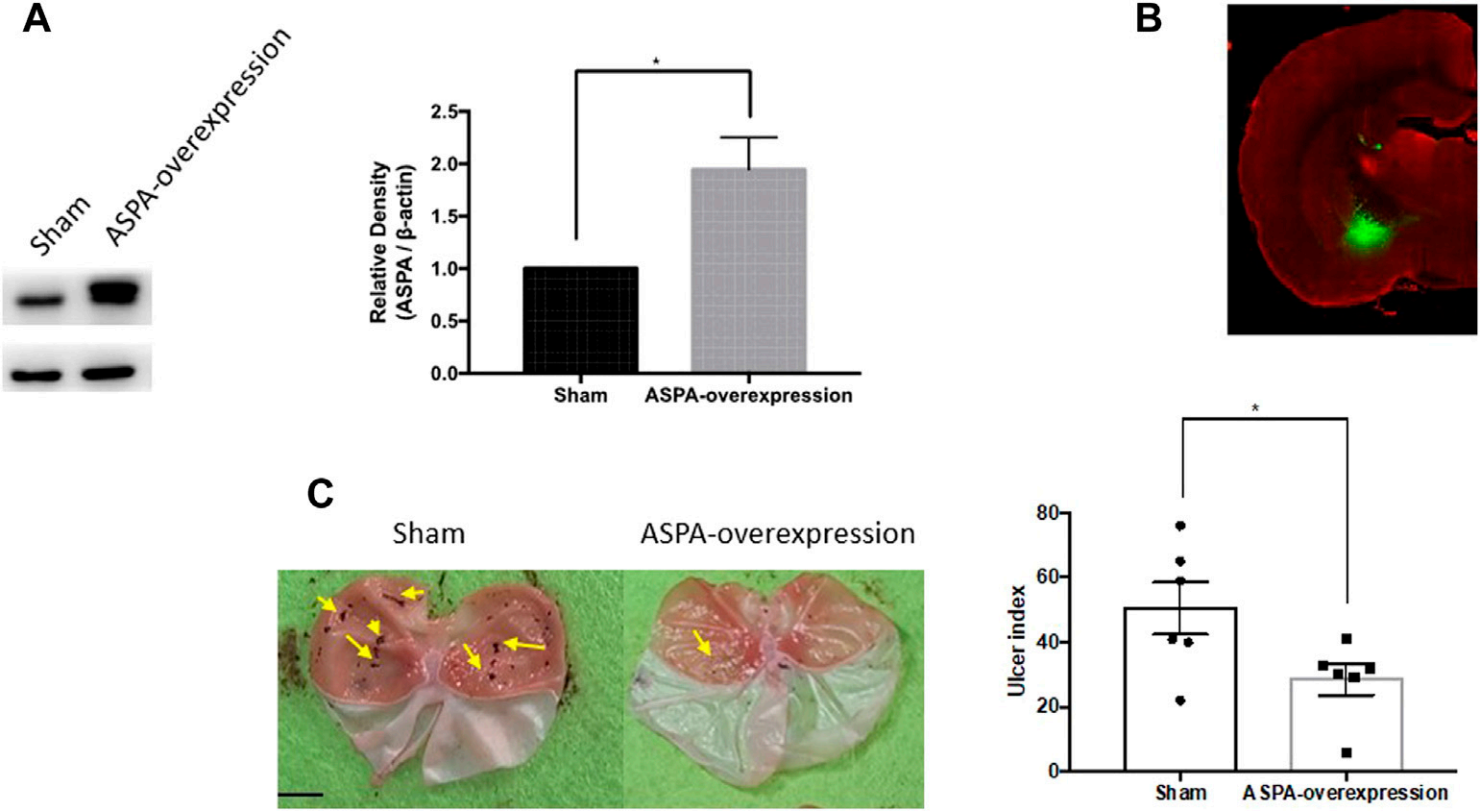
Overexpression of NAA hydrolase ASPA in CeA reduced gastric ulcer caused by WIRS. **(A)** Verification of intervention results by Western blot. (*n* = 3 per group). **(B)** Representative image of injection sites (CeA) in rat brain. **(C)** Overexpression of ASPA in CeA reduced gastric ulcer caused by water immersion (*n* = 6). The arrow indicates the ulcer position. Scar bar, 10 mm. Values are expressed as means ± SEM. *p<0.05, compared with sham viral group.

To confirm the role of ASPA in the CeA in the WIRS-induced gastric ulcer, we overexpressed ASPA in the bilateral CeA and tested the effects in the WIRS model. The ASPA level was upregulated 3 weeks after injecting rAAV-EF1a-ASPA-EGFP-WPRE-pA into the CeA (Figures 4A,B). The overexpression of ASPA in CeA alleviated WIRS-induced gastric ulcer significantly (Figure 4C, *p* < 0.05).

**FIGURE 5:**
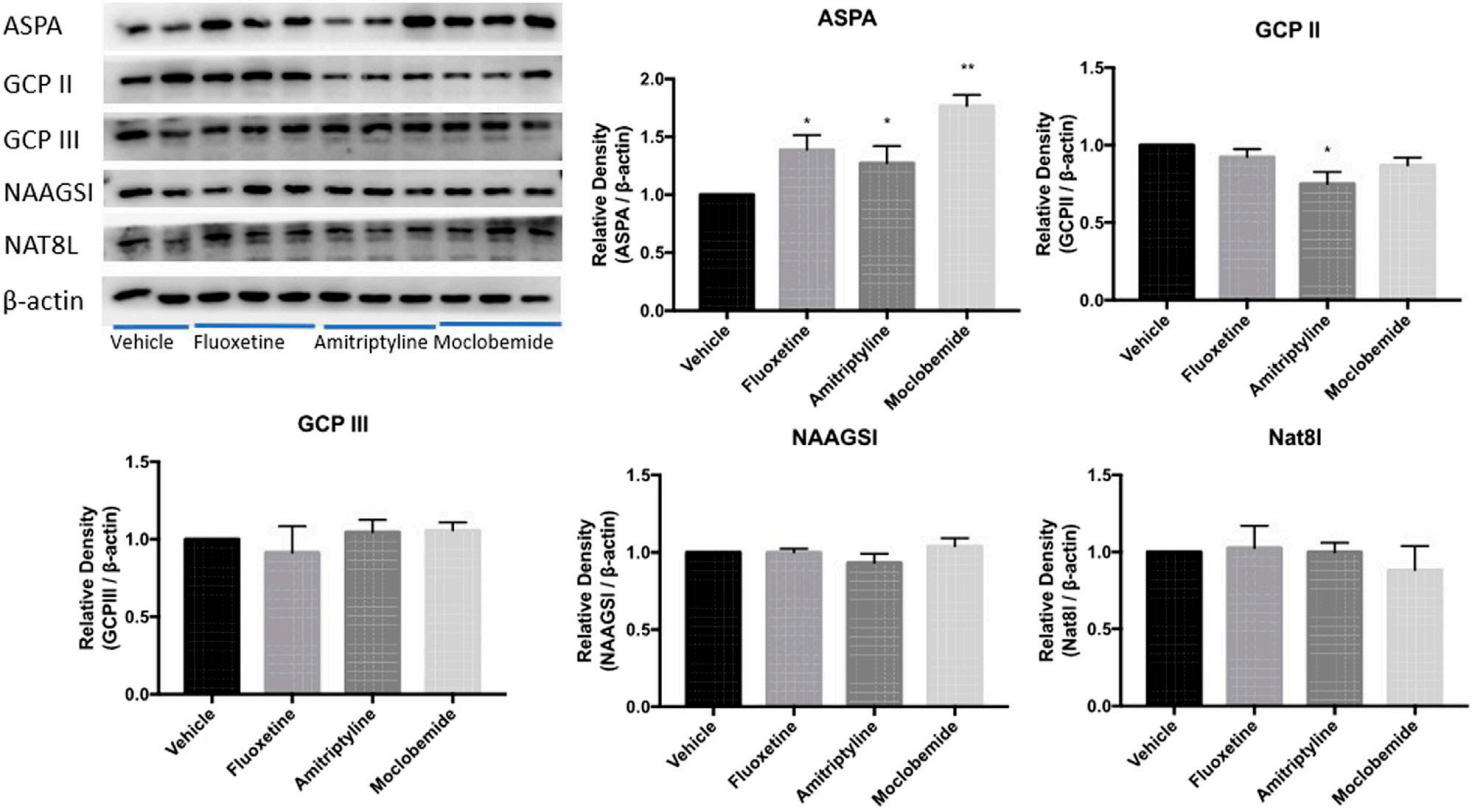
Effects of other three kinds of monoamine-based antidepressants on NAA- related enzyme expression in CeA in rats exposed to 0.5 h of WIRS. Pretreatment with 10 mg/kg fluoxetine, 10 mg/kg amitriptyline, and 20 mg/kg moclobemide all increased ASPA levels in the condition of WIRS for 0.5 h, which were consistent with duloxetine. Amitriptyline also reduced GCPII level. ASPA/GCP II/GCP III/NAAGSI/Nat8l was normalized to β-actin. Values are expressed as means ± SEM. *p < 0.05, **p < 0.01, compared with vehicle group. (*n* = 4 in fluoxetine group, *n* = 5 in moclobemide group, *n* = 6 in vehicle and amitriptyline group)

To investigate whether it was common in the regulation of the five enzymes by the other monoamine-based antidepressants including MAOs, TCAs, and SSRIs, we performed Western blotting to examine the expression of ASPA/GCP II/GCP III/NAAGSI/Nat8l in the CeA of rats pretreated with moclobemide, amitriptyline, and fluoxetine in the WIRS model at 0.5 h. The results showed that ASPA levels in the CeA increased significantly in all the three groups compared with the vehicle group (Figure 5, *p* < 0.05, *p* < 0.05, *p* < 0.01). Besides, GCP II was decreased in the amitriptyline treatment group (*p* < 0.05). Thus far, our data suggested that monoamine-based antidepressants prevented gastric mucosa damage from WIRS by upregulating the ASPA expression in CeA to reduce NAA.

## Discussion

The data presented here showed that pretreatment with duloxetine significantly reduced NAA/Cre in the CeA after 6 h of WIRS, and the change of NAA/Cre in CeA was negatively correlated with the gastric ulcer index. Moreover, the intra-CeA infusion of NAA worsened gastric mucosa damage. The upregulation of ASPA may enhance the hydrolysis of NAA, leading to a decrease in NAA, which may be one of the common mechanisms underlying gastroprotective effects from WIRS by monoamine-based antidepressants.

To the best of our knowledge, this is the first study that applied ^1^H-MRS to investigate the metabolic effects of the drug playing a gastroprotective role in WIRS-treated rats. The application of MRS *in vivo* has advanced our understanding of change of neurometabolites in a specified brain region during the protective process of duloxetine against WIRS. Creatine can be used as a marker of energy metabolism in the brain, and the concentration of total creatine is considered relatively stable throughout the brain. So creatine is often used as an internal reference to measure the content of other metabolites in many spectral studies (Rackayova, et al., 2017; Maddock and Buonocore 2012). NAA/creatine in CeA did not change 30 min after duloxetine administration, and it was not associated with the gastric ulcer index after experiencing 6 h of WIRS, while the greater decrease of NAA/creatine in the CeA was related to a more serious gastric mucosa damage induced by WIRS. This suggested that it was the change of NAA/Cre other than baseline NAA/creatine in CeA that could be used as a marker to predict the severity of gastric mucosa damage induced by WIRS. Pretreatment with duloxetine lowered NAA/creatine in the CeA paralleled by the gastroprotective effect, and the intra-CeA infusion of NAA worsened the gastric mucosa injury, which suggested that the reduction of NAA in CeA may be one of the ways that duloxetine prevented stomach injury from WIRS.

NAA is the second most abundant metabolite in the brain after glutamate and appears in a prominent peak in ^1^H-MRS of the brain. NAA is synthesized in the mitochondria of neurons and is directly associated with mitochondrial function (Patel and Clark 1979). The signal of NAA is considered to reflect the density and vitality of neurons or the integrity of neuronal mitochondria (Paslakis, et al., 2014). A decrease in NAA/creatine in CeA may be a marker of reversible neuronal dysfunction. CeA has received considerable attention as a key node for stress integration and is differentially activated by homeostatic disruption and systemic, but not psychogenic, stressors. Physiological studies have demonstrated that the electrical stimulation of CeA increased the susceptibility to stress ulcer formation, while bilateral lesions in the CeA reduced the severity of stress ulceration (Henke 1988; Henke, et al., 1991). Moreover, WIRS for 3 h induced increases in neuronal activity and altered the firing pattern in CeA, as well as activated corticotrophin-releasing hormone neurons in the CeA, which could be involved in the formation of stress-induced gastric ulceration (He, et al., 2018)^.^ Therefore, a prophylactic injection of duloxetine may protect from stress ulcer formation *via* inhibiting the neuronal activity of CeA.

NAA is synthesized *via* the N-terminal acetylation of aspartate amino acid from aspartate and acetyl-coenzyme A by Nat8l in neurons, and in some neurons, a portion of NAA is converted into N-acetylaspartylglutamate (NAAG) by NAAG synthetase I (NAAGSI) (Becker, et al., 2010). Released NAAG can be degraded by glutamate carboxypeptidase II (GCP II) or GCP III, which is a membrane-bound enzyme mainly expressed by astrocytes, liberating NAA and glutamate (Lyeth 2015; Neale and Yamamoto 2020). In oligodendrocytes (OLs), NAA can be degraded by ASPA to generate aspartate and acetate (Moffett, et al., 2011). As time goes on, the effects of WIRS on the expression of five kinds of NAA-related enzymes are different. All rats exposed to WIRS for 6 h have increased NAAGSⅠ expression in CeA regardless of pretreatment with duloxetine, which may be the effect of the longer WIRS process. WIRS for 0.5 h reduced the expression of ASPA in CeA, which may decrease the hydrolysis of NAA and then increase the content of NAA. The following downregulating GCPⅡ and NAT8L expression in the CeA after WIRS for 3 h may be a negative feedback mechanism of cutting down NAA synthesis from both neurons and astrocytes, which may offset the increase of NAA derived from downregulating ASPA after 0.5 h WIRS. It can explain why there was no significant change of NAA in CeA after 6 h of WIRS. Reduced ASPA in CeA by 0.5 h of WIRS was increased by pretreated 20 mg/kg duloxetine, which would maintain the contents of NAA in CeA and bring the increase of NAAGSⅠ expression forward. The increased expression of NAAGSⅠ in the CeA from 3 h of WIRS led to a decrease of NAA ultimately. Although GCP Ⅲ was marginally increased by 20 mg/kg duloxetine, the lower expression level and NAAG-hydrolyzing activity (Neale, et al., 2011; Hlouchová, et al., 2007) may make it affect the level of NAA slightly. Furthermore, the overexpression of ASPA in CeA alleviated gastric ulceration induced by WIRS, which suggested that ASPA may be a crucial enzyme regulated by duloxetine to decrease NAA, and it was also one of the common molecular mechanisms involved in the antiulcer effects of monoamine-based antidepressants. In a word, pretreatment with monoamine-based antidepressants seems to enhance the resilience to stress ulceration *via* increasing the ASPA of OLs to reduce NAA in the CeA.

Chronic stress has been shown to downregulate the gene transcription or expression of OLs in amygdala, especially in CeA. In an important translational study, about 30–40 genes were altered in expression in both species in terms of the amygdala transcriptome of mice exposed to chronic stress and depressed human subjects, and 30% of these genes that are exclusively related to the OL structure and function were all downregulated (Sibille, et al., 2009). Furthermore, chronic social stress has been shown to downregulate OL-enriched gene expression in the prefrontal cortex, amygdala, and hippocampus, and CeA was more responsive to chronic stress in terms of the number of downregulated OL genes, in which the ASPA gene expression in stressed mice showed 1.46-fold downregulation (Cathomas, et al., 2019). Moreover, CD4+T cell-derived xanthine acts on OLs in the left amygdala *via* adenosine receptor A1 and triggers the onset of anxiety by chronic physical stress (Fan, et al., 2019). Our study for the first time presented that WIRS just for 0.5 h, following 48 h of fasting, can decrease the expression of ASPA in OLs, and pretreatment with multiple monoamine-based antidepressants maintained the level of ASPA in the CeA. It seemed that the expression of ASPA in CeA was transiently downregulated by a single stress, while it did not return to the control level after chronic stress. ASPA is predominantly expressed in mature OLs where it functions as a homodimer to cleave NAA, releasing acetate that can be used for myelin lipid synthesis (Baslow, et al., 1999; Moore, et al., 2003; Jolly, et al., 2016). The lack of ASPA activity results in the accumulation of NAA and is tightly linked to the Canavan disease, which is a fatal neurodegenerative disorder genetically linked to polymorphisms in the ASPA gene such as substitution C152W (Gersing, et al., 2021; Madhavarao, et al., 2005). OLs’ list of known functions extends beyond axon myelination and now includes the direct modulation of neuronal function (de Hoz and Simons 2015; Jha and Morrison 2020). Our results provided another possible way of crosstalking between glia and neurons *via* NAA to be involved in the response to stress and the protective effect of monoamine-based antidepressants.

Glucocorticoid (GC) is released in response to stress as the end product of the HPA axis and known to readily cross the blood–brain barrier to affect brain processing. Corticosteroids exert their actions upon binding the intracellular mineralocorticoid receptors (MRs) and glucocorticoid receptors (GRs) that regulate gene expression in tissue. OLs in amygdala contain a high density of GRs, and MRs are abundantly expressed in CeA (Arriza, et al., 1988; Matsusue, et al., 2014). Moreover, increased GC levels regulate amygdala activity (Henckens, et al., 2012). However, whether WIRS downregulated ASPA expression through GC-GR/GC-MR interactions and what the detailed mechanism was remained to be further explored.

Our previous study has shown that the DUOX2 isoform of NADPH oxidases in the gastric mucosa was a common modulator in the preventative effects of monoamine-based antidepressants on WIRS- and indomethacin-induced gastric ulcers (Cao, et al., 2020). The present study provided a central joint molecular target implicated in the gastroprotective effects on WIRS of this kind of drug, which may coordinate with the peripheral actions. Furthermore, our research revealed a novel mechanism underlying monoamine-based antidepressants, which may be used as the potential target for depression treatment.

Collectively, pretreatment with monoamine-based antidepressants exerted gastroprotective effects against stress by increasing ASPA to hydrolyze NAA in CeA, and ASPA was a shared target for monoamine modulatory antidepressants. Our work provides another mechanism of this type of drugs more than monoamine regulation.

## Data Availability

The datasets presented in this study can be found in online repositories. The names of the repository/repositories and accession number(s) can be found below: GenBank, NM_024399.1.
